# Knee arthrodesis as last resort for persistent knee joint infections: Comparison of extramedullary and intramedullary treatment

**DOI:** 10.1007/s00132-020-03939-z

**Published:** 2020-07-14

**Authors:** Dirk Zajonz, Benedikt Zimmerlich, Robert Möbius, Melanie Edel, Johanna Przybyl, Andreas Höch, Johannes K. M. Fakler, Andreas Roth, Mohamed Ghanem

**Affiliations:** 1 Department of Orthopaedics, Trauma and Plastic Surgery, University Hospital Leipzig, Liebigstraße 20, 04103 Leipzig, Germany; 2 Center for Research on Musculoskeletal Systems, ZESBO, Semmelweisstraße 14, 04103 Leipzig, Germany; 3 Department of Orthopaedic Surgery, Traumatology and Reconstructive Surgery, Zeisigwald Hospital Chemnitz, Zeisigwaldstraße 101, 09130 Chemnitz, Germany; 4 Clinic for Orthopaedics and Trauma Surgery, Sana Kliniken Leipziger Land, Rudolf-Virchow-Straße 2, 04552 Borna, Germany

**Keywords:** Retrospective study, Total knee arthroplasty, Periprosthetic infection, Debridement, Gonarthrosis, Retrospektive Studie, Knietotalendoprothese, Periprothetische Infektion, Débridement, Gonarthrose

## Abstract

**Background:**

Knee joint arthrodesis is an established treatment for periprosthetic infections (PPI) providing stability and pain relief. In this study the outcome after arthrodesis of the knee joint for persistent infections was compared and evaluated depending on the surgical procedure (intramedullary vs. extramedullary).

**Material and methods:**

In a retrospective case analysis, all patients who underwent knee joint arthrodesis between 1 January 2010 and 31 December 2016 were identified and divided into two groups: IMA and EMA. All patients were examined clinically and radiologically and the patient files were evaluated. In addition, the FIM score, the LEFS, the WHOQOL-BREF and NRS were evaluated.

**Results:**

The median LEFS score for the IMA group was 26 points and in the EMA group 2 points (*p* = 0.03). The IMA patients showed a median pain scale at rest of 0 and during exercise of 2. The EMA group recorded a pain scale of 3 at rest and 5 during exercise (*p* = 0.28 at rest; *p* = 0.43 during exercise). In the IMA group the median postsurgical leg length difference was −2.0 cm and −2.5 cm in the EMA group (*p* = 0.31). At the end of the follow-up examinations, the FIM score of patients in the IMA group was 74.5 points and 22 points in the EMA group (*p* = 0.07).

**Conclusion:**

The study showed that no arthrodesis procedure is obviously superior with respect to the postoperative outcome. The IMA combines advantages especially in the early phase after surgery in terms of function as well as patient comfort and is therefore currently the procedure of choice. The attending physician should be familiar with the advantages and disadvantages of the various procedures in order to be able to make an individual decision and thus maximize the chance of treatment success.

## Abstract (de)

**Hintergrund:**

Die Kniegelenkarthrodese ist eine etablierte Behandlung von periprothetischen Infektionen (PPI), die Stabilität und Schmerzlinderung bietet. In dieser Studie wurde das Ergebnis nach einer Arthrodese des Kniegelenks bei persistierenden Infektionen verglichen und in Abhängigkeit vom Operationsverfahren (intramedullär vs. extramedullär) bewertet.

**Material und Methoden:**

In einer retrospektiven Fallanalyse wurden alle Patienten, die sich zwischen 1. Januar 2010 und 31. Dezember 2016 einer Kniegelenkarthrodese unterzogen haben, identifiziert und in 2 Gruppen eingeteilt: IMA und EMA. Alle Patienten wurden klinisch und radiologisch untersucht und die Patientenakten ausgewertet. Darüber hinaus wurden der FIM-Score, der LEFS, der WHOQOL-BREF und der NRS ausgewertet.

**Ergebnisse:**

Der mediane LEFS-Score betrug in der IMA-Gruppe 26 Punkte und in der EMA-Gruppe 2 Punkte (*p* = 0,03). Die IMA-Patienten zeigten eine mediane Schmerzskala in Ruhe von 0 und während der Belastung von 2, die EMA-Gruppe eine Schmerzskala von 3 in Ruhe und 5 während der Belastung (*p* = 0,28 in Ruhe; *p* = 0,43 während der Belastung). In der IMA-Gruppe betrug der mediane postoperative Beinlängenunterschied –2,0 cm und –2,5 cm in der EMA-Gruppe (*p* = 0,31). Am Ende der Nachuntersuchungen lag der FIM-Score der Patienten in der IMA-Gruppe bei 74,5 Punkten und in der EMA-Gruppe bei 22 Punkten (*p* = 0,07).

**Schlussfolgerung:**

Die Studie zeigte, dass kein Arthrodeseverfahren im Hinblick auf das postoperative Ergebnis klar überlegen ist. Die IMA vereint insbesondere in der frühen Phase nach der Operation Vorteile sowohl hinsichtlich der Funktion als auch des Patientenkomforts und ist daher derzeit das Verfahren der Wahl. Der behandelnde Arzt sollte mit den Vor- und Nachteilen der verschiedenen Verfahren vertraut sein, um eine individuelle Entscheidung treffen zu können und so die Chance auf einen Therapieerfolg zu maximieren.

## Introduction

The implantation of total knee arthroplasty (TKA) is most commonly performed in pronounced primary and secondary gonarthrosis after exhausting all non-surgical treatment options [1]. This is associated with a steady increase in primary arthroplasty as well as in the number of revision arthroplasty after TKA in industrialized countries [2, 3]. Despite optimizing surgical strategies and the development of prophylactic antibiotic treatment, periprosthetic infection (PPI) remains one of the most feared complications with a prevalence of 1–3% [4–6]. Particularly in complicated and chronic periprosthetic knee joint infections, the 2‑stage procedure is the gold standard for the treatment of a PPI [7, 8]. The infected implants are removed, radical debridement is performed and a cement spacer containing an antibiotic is inserted [7, 8]. In addition, a systemic antibiotic treatment is carried out [9]. Once the infection has healed with sufficient probability, a reimplantation can be performed.

Despite a 2-stage approach, the frequency of persistent infections or recurrences is 9–12% [10, 11]. In such cases, knee joint arthrodesis is an established treatment alternative for rehabilitation of the patient following a PPI. It provides stability and pain relief in the knee joint [12, 13]. In addition to persistent PPI, which is by far the most common indication for arthrodesis, there are a number of other pathological conditions that may require knee joint arthrodesis. Examples are septic arthritis, osteomyelitis and an insufficiency of the stretching apparatus with accompanying joint infection [27]. Knee joint arthrodesis can be performed with an intramedullary nail, a tibiofemoral plate osteosynthesis or an external fixator [14–16]. The selection of the appropriate procedure depends on various factors. In addition to the soft tissue situation, the presence of bone defects and insertion of further endoprostheses or osteosynthesis materials in the adjacent joints, surgeon experience and patient preference also play a decisive role [17, 18]. The various procedures have individual advantages and disadvantages. It has been described that IMA, with good reconstruction of the leg length provides more stability, and allows the patient to bear weight on the injured knee in a much faster manner [14, 19]; however, in the case of a recurrence of infection retreatment strategies are often limited [20]. The use of EMA with one or two osteosynthesis plates is usually associated with a considerable shortening of the leg, but offers the possibility of material removal in recurrences after consolidation [21, 22]. External fixation arthrodesis is a proven surgical method in cases with poor soft tissues but is generally less well accepted by patients [16, 23].

**Fig. 1:**
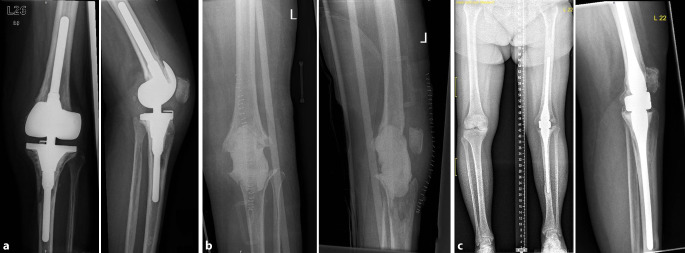
Anteroposterior (*left*) and lateral (*right*) X‑rays of the knee (**a**) of a 72-year-old female patient with left-sided periprosthetic infection after revision total knee arthroplasty, **b** after resection arthroplasty and placement of a temporary cement spacer and **c** healed arthrodesis using a modular intramedullary nailing system (courtesy of the Department of Diagnostic and Interventional Radiology, University Hospital of Leipzig, all rights reserved)

**Fig. 2:**
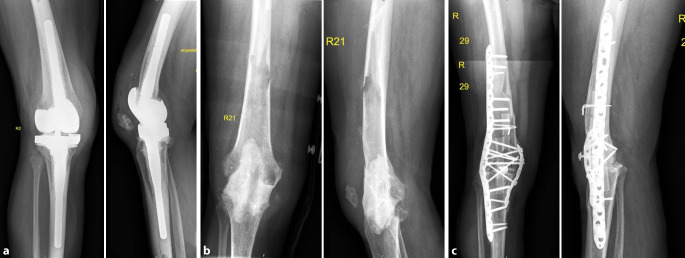
Anteroposterior (*left*) and lateral (*right*) X‑rays of the knee (**a**) of an 80-year-old female patient with right-sided periprosthetic infection after revision total knee arthroplasty, **b** after resection arthroplasty and placement of a temporary cement spacer and **c** healed arthrodesis using two compression plates and strut grafts (courtesy of the Department of Diagnostic and Interventional Radiology, University Hospital of Leipzig, all rights reserved)

In this study, the outcome after arthrodesis of the knee joint in persistent infections is compared and evaluated in our own patient population depending on the surgical procedure (intramedullary vs. extramedullary). Examples of the IMA (Fig. 1) and EMA (Fig. 2) procedures are given showing the different stages and outcomes.

## Material and methods

A positive vote of the university ethics committee was obtained (390/18 -ek) prior to conducting this study. All methods and investigations were performed in accordance with the relevant guidelines and regulations for conducting clinical trials.

A retrospective case analysis of the patient archiving system (IS‑H SAP; Siemens AG Healthcare Sector, Erlangen, Germany) identified all patients who underwent knee joint arthrodesis between 1 January 2010 and 31 December 2016. Patients were divided into two groups, depending on the method used: intramedullary arthrodesis (IMA) and extramedullary arthrodesis (EMA). All patients were examined clinically and radiologically, and the patient files were evaluated. In addition to patient-specific factors such as age, sex and indication, details of the procedure such as duration of surgery, implant manufacturer, type of care and in particular complications, were compared. For the follow-up examination, a standardized whole leg X‑ray scan in a standing position was performed and evaluated with a scale. The difference in leg length (cm) was determined clinically and radiologically. In addition, patients assessed whether bearing weight on the extremity was possible from 100% (subjectively possible) to 0% (subjectively impossible). Pain in the operated leg was also assessed on the numeric rating scale (NRS) from 0 (no pain) to 10 (worst imaginable pain) both at rest and during exercise. Furthermore, patients were asked about the possible use of walking aids and the maximum walking distance. In addition, various scores such as the functional independence measure (FIM) score, which measures functional limitations of patients on the basis of 13 characteristics, were collected for follow-up examination [24]. A uniform 7-point scale (1–7 points) was used for all characteristics. This index provides a total number of points between 13 and 91. Furthermore, the lower extremity functional scale (LEFS) was used to measure the disability after injury and diseases of the lower extremities, and to objectively map the therapeutic process [25]. The LEFS comprises 20 items for different activities, which can be rated with 0 points (extreme difficulty/unable to perform activity) to 4 points (can be performed with no difficulties). The WHO quality of life short version (WHOQOL-BREF) was also used. This is a patient reported outcome instrument tool use to evaluate the patients’ global health status, independent of the nature of the disease, in 4 health domains (physical health, psychological health, social life and environment), and 24 different aspects of these domains [26]. All data were digitized and evaluated using Microsoft Excel Version 2013 (Microsoft Corporation, Redmond, WA, USA) and SPSS Version 24.0 (IBM, Chicago, IL, USA). *P*-values of 0.05 or less were considered as statistically significant.

## Results

In the evaluation period from 1 January 2010 to 31 December 2016, 25 cases with appropriate care were recorded and assigned to the 2 groups IMA (*n* = 18) and EMA by means of tibiofemoral plate arthrodesis (*n* = 7). The median follow-up time of the IMA was 51 months (range 10–82 months). In the EMA group, the adjusted follow-up time was 28 months (range 2–44 months), which is a significant difference compared to the IMA (*p* = 0.02). The median age of the patients with IMA at the time of surgery was 76.6 years (range 60.6–88.5 years). The median age of those in the EMA group was 60.6 years (range 55.0–80.3 years, *p* = 0.04). All EMA (*n* = 7) were carried out without cement but in the IMA group 10/18 were uncemented (56%) and 8/18 (44%) cemented nail arthrodesis; however, the different forms of IMA did not statistically differ in a significant way in any of the aspects examined, which is why only the entire IMA is considered (Table 1).

**Table 1:** Comparison of the examination groups intramedullary arthrodesis vs. extramedullary plate arthrodesis with respect to number (absolute, %) of female subjects, median age, follow-up and surgery time (min–max), cement augmentation and implant specifics

	Intramedullary arthrodesis (IMA)	Extramedullary plate arthrodesis (EMA)	Statistical significance (α = 0.05)
Female	61% (11/18)	43% (3/7)	0.41
Median age at time of surgery in years (range)	76.7 (60.6–88.5)	60.6 (55.0–80.3)	**0.04**
Median follow-up examination in months (range)	51 (10–82)	28 (2–44)	**0.02**
Cement augmentation	44% (8/18)	N/A	–
Median surgery time in minutes (range)	167 (91–417)	191 (151–296)	0.42
Number of implants used:			
Modular nail system	88% (16/18)	N/A	–
Long arthrodesis nail	6% (1/18)	N/A	–
Cemented carbon rod	6 (1/18)	N/A	–
2 Internal fixation	N/A	57% (4/7)	–
2 Internal fixation + strut graft	N/A	29% (2/7)	–
1 Internal fixation + 2 crossed GFI screws	N/A	14% (1/7)	–

Statistically significant values are highlighted in bold

*N/A* not available, *GFI* Large Fragment Interlocking Screws

The median time of the procedure for IMA was 167 min (range 91–417 min), and 191 min (range 151–296 min) for EMA (*p* = 0.42). Infections after surgery were present in 6/18 (33%) in the IMA group, whereas infections were present in 2/7 (29%) in the EMA group (*p* = 0.82). Bone consolidation was demonstrated in 6/7 (86%) cases (1 case without sufficient time to follow-up examination) and no pseudarthroses were observed. In the IMA group, in 6/18 (33%) cases the nail became loosened, and 4 of the 6 loosened nails were caused by a pathogen detected in the arthrodesis area. Hence, these cases were classified as septic nail loosening. Looking at all the implant-related complications (IMA: persistent infection, septic and aseptic loosening, peri-implant bone fracture, amputation; EMA: persistent infection, material fracture, psychological stress caused by arthrodesis and the desire to amputate the affected limb), 10/18 IMA cases (56%) were found compared to 3/7 EMA cases (*p* = 0.39). In the IMA group, the median difference was −2.0 cm (range –13.0 to +2.5 cm). In the EMA group, the difference was −2.5 cm (range: −25.0–−2.0) (*p* = 0.31) (Table 2).

**Table 2:** Comparison of the examination groups intramedullary arthrodesis vs. extramedullary plate arthrodesis with respect to complications (absolute, %)

	Intramedullary arthrodesis (IMA)	Extramedullary plate arthrodesis (EMA)	Statistical significance (α = 0.05)
Infection persistence or reinfection	33% (6/18)	29% (2/7)	0.82
Material loosening	33% (6/18)	N/A	–
Aseptic	11% (2/18)	N/A	–
Septic	22% (4/18)	N/A	–
Pseudarthrosis	N/A	0% (0/7)	–
Other			
Peri-implant femoral fracture	17% (3/18)	N/A	–
Amputation	6% (1/18)	N/A	–
Desire for amputation with intact arthrodesis	N/A	14% (1/7)	–
Material fracture	N/A	14% (1/7)	–
Soft tissue defect	N/A	14% (1/7)	–

Walking aids such as crutches, walkers and rollators were required in 14/18 cases in the IMA group (78%), 3 out of 18 patients (17%) had to use a wheelchair but 1 out of 18 patients (6%) was able to walk without any walking aid. In 4 out of 7 cases in the EMA group (57%), patients needed walking aids, whereas 1 out of 7 (14%) had to use a wheelchair and 2/7 (28%) were bedridden. The IMA patients showed a median pain scale (numeric rating scale) at rest of 0 (range 0–5) and during exercise of 2 (range 0–6). The EMA group recorded a pain scale of 3 (0–6) at rest and 5 (0–8) during exercise (*p* = 0.28 at rest; *p* = 0.43 during exercise). Weight-bearing (0–100% of the patient’s body weight) on the operated limb was possible at 84% within the IMA group (range: 20–100%), whereas the EMA patients were only able to bear weight on the limb at 60% (range 0–100%, *p* = 0.02; statistically significant). The median maximum walking distance of patients in the IMA group was 100 m (range 0–7000 m) and patients in the EMA group were able to walk 25 m (range 0–200 m) (*p* = 0.21).

The median LEFS score for the IMA group was 26 points (range 15–53 points), and the EMA group achieved 2 points (range 2–2, *p* = 0.03). An immediately preoperative FIM with a median of 91 points (range 73–91 points) in the IMA group was compared with 52 points (range 30–74 points, *p* = 0.06). On discharge from hospital, patients in the IMA group reached 66 FIM points (range 73–91 points). Patients in the EMA group reached 22 points (range 18–26 points, *p* = 0.04). At the end of the follow-up examinations, the FIM score of patients in the IMA group was 74.5 points (range: 25–91 points), and 22 points (range: 18–26 points) in the EMA group (*p* = 0.07). A WHOQOL-BREF survey was only possible among IMA patients. The median score for the domain physical health was 50 points at the time of the follow-up examination (range 31–71 points), for the domain of psychological health the median score was 75 points (range 44–81 points), for the domain social relations 81 points (range 44–81 points), and environment 63 points (range 38–81 points). In the EMA group, no patient data were collected for the WHOQOL-BREF survey.

## Discussion

Persistent infections of the knee joint represents a major therapeutic challenge, in which, after the failure of joint-preserving therapeutic attempts, an arthrodesis of the affected joint has proven to be one of the most reliable surgical treatments for definitive healing of infection while preserving the extremity [15].

The procedures frequently described and compared in the literature are intramedullary nail arthrodesis and EMA using an external fixator [28, 29]. In addition to the previously mentioned nail arthrodesis, the tibiofemoral (double) plate arthrodesis has been used in our patients. The external fixator has its place in the arthrodesis of the knee joint but this method requires a good patient compliance, daily care of pins, mindfulness and acceptance of the outer frame and the associated limited comfort, as bearing full weight on the limb is achieved only slowly. Furthermore, there is a risk of loosening of pins and an infection of pin sites [28], which is why an external fixator was not used in our patients. The EMA using internal fixation or arthrodesis plates shows a comparable, if not better, fusion of bones compared to external fixator, with significantly greater comfort for the patient. Nevertheless, only a few studies have been carried out with the plate EMA. The number of publications comparing this technique with IMA is even smaller [30, 31].

Regardless of the method used, the primary therapeutic goals are to cure the infection while maintaining the functionality of the extremity in the best possible way. In 2018 Balato et al. were able to show in a systematic review and meta-analysis of 26 publications that in IMA the rate of reinfection and persistence of infection was 13.3% (422 cases studied), while other studies reported rates between 0% (19 investigated cases in Letartre et al. 2009) and 50% (26 cases studied by Röhner et al. 2015) [32–34]. In contrast, our study showed a proven rate of persistent infections or reinfections of 33% (18 cases studied). Differences in the individual publications can be explained by the large heterogeneity of the patients treated. Moreover, an inconsistent definition of the terms reinfection and infection control is probably responsible for the discrepancies. Especially older publications with high rates of infection eradication should be critically considered [34]. With respect to infection control, there are only few data on EMA using internal fixators. Nichols et al. reported persistence of infections in 14% (7 cases studied), other studies and case reports ranged from 0% (3 cases studied in Kuo et al.) to 33% (3 cases studied by Van Rensch et al.) [35–37]. In our patient population, a persistent joint infection was found in 29% (7 cases examined) after internal fixator EMA. If one compares the therapeutic success in terms of infect eradication in our data, 33% persistent infections are found within the IMA group vs. 29% within the EMA group, which is not a significant difference (*p* = 0.82). We are not aware of any publications investigating both arthrodesis methods with respect to this question. Consequently, a classifying comparison is not possible here.

For many years, scientists agreed on the superiority of IMA in terms of better bone consolidation and fusion in the area around the arthrodesis; however, Balato et al. came to the conclusion in their major review that there was no significant difference in bone consolidation and/or bone fusion [32]. In our patient population, safe bony consolidation of the EMA was also demonstrated in all cases with a sufficiently long time before the follow-up examination, which also largely corresponds with the results of Nichols et al. and Robinson et al. [35, 38]. Contrary to this, the rates of material loosening in the IMA patients, which was at 33% (18 cases examined), were relatively high. One explanation for this could be that 4 of the 6 cases observed with loosening were of septic origin. This means that not the IMA procedure itself caused the loosening of the nail but the unsuccessful infection eradication. A comparative analysis of bone consolidation between EMA and IMA is not possible in our patient population, because modular short arthrodesis nails were used, which means that bone contact with consecutive dilation is not necessary here.

The overview of all implant-related complications does not show any significant difference when comparing the procedures; however, it can be seen that regardless of the procedure used there is a high risk of complications (56% for IMA vs. 43% for EMA), as other authors confirmed (46.5% for Schwarzkopf et al., 41% for Leroux et al., 65% in Robinson et al.) [38–40].

Many other investigated aspects show no statistically significant difference between IMA and EMA; however, a tendency in favor of IMA can be seen in a large number of the study criteria. The postoperative leg length difference is smaller, and patients reported that they suffered less pain both at rest and during exercise. Moreover, they required less help with mobilization, and the subjectively assessed possibility to bear weight on the limb as well as their maximum walking distance was better. These tendencies were also reflected in the collected function scores. The LEFS score showed a significant difference in favor of the IMA. The FIM score was also higher at all three times of the survey; however, a comparison of the scores between the two groups of patients and the significant differences found must be viewed critically, since the only useful data come from only two patients of the EMA group, who already had a worse (everyday) function before surgery. Nonetheless, the tendencies described correspond with the results of other publications (cf. Bierwagen et al., Robinson et al.) [28, 38].

## Limitations

The main limitation of our study is the limited number of cases; however, most comparable publications dealing with the subject of knee joint arthrodesis include a similar number of patients. Therefore, it is difficult to obtain statistically valid findings, although tendencies are recognizable. Moreover, the study examined an extremely heterogeneous patient population. Especially the statistically significant difference in age and check-up time impairs the comparability of the two groups. The individual treatment (surgical technique, implants, follow-up treatment, etc.) was subject to certain differences within the individual groups. In addition, the retrospective study design prevented the collection of more comprehensive and complete data.

## Conclusion

Our study showed that there is no obviously superior arthrodesis procedure with respect to the postoperative outcome. Therefore, a general recommendation for a specific procedure cannot be made. The intramedullary method combines advantages especially in the early phase after surgery in terms of function and patient comfort (rapid full weight-bearing on the extremity, mobilization, lower pain level, possibility of better correction of the leg length. difference with modular technique) and is therefore currently the procedure of choice; however, there are relatively high complication rates in IMA. In cases where an intramedullary technique does not seem possible, EMA using internal fixators is a sensible alternative. Especially with respect to infection recurrence, an extramedullary technique offers the possibility of material removal. In addition, in the case of a repeated infection, unlike in nail arthrodesis, there is no risk of septic loosening of the arthrodesis after bony reconstruction.

Infections of the knee joint always involve complex treatment decisions. The attending physician should therefore be familiar with all the advantages and disadvantages of the various procedures in order to be able to make an individual decision and thus maximize the chance of therapeutic success.

## Abbreviations

EMA: Extramedullary arthrodesis
FIM: Functional independence measure
IMA: Intramedullary arthrodesis
LEFS: Lower extremity functional scale
NRS: Numeric rating scale
PPI: Periprosthetic infection
TKA: Total knee arthroplasty
WHOQOL-BREF: World Health Organization quality of life short version
